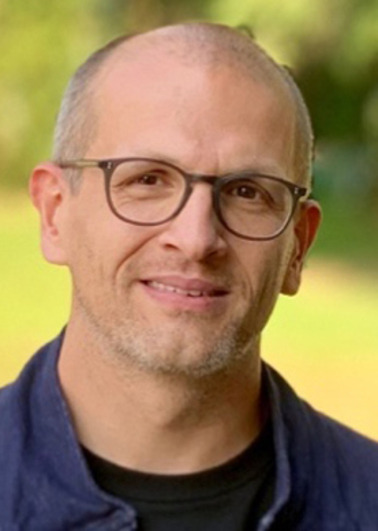# Recent Developments and Trends in Asymmetric Organocatalysis

**DOI:** 10.1002/ejoc.202200950

**Published:** 2022-10-17

**Authors:** Olga García Mancheño, Mario Waser

**Affiliations:** ^1^ Organic Chemistry Institute University of Münster Corrensstrasse 36 48149 Münster Germany; ^2^ Institute of Organic Chemistry Johannes Kepler University Linz Altenbergerstrasse 69 4040 Linz Austria

**Keywords:** Catalyst design, Method development, Organocatalysis, Stereochemistry

## Abstract

Asymmetric organocatalysis has experienced a long and spectacular way since the early reports over a century ago by von Liebig, Knoevenagel and Bredig, showing that small (chiral) organic molecules can catalyze (asymmetric) reactions. This was followed by impressive first highly enantioselective reports in the second half of the last century, until the hype initiated in 2000 by the milestone publications of MacMillan and List, which finally culminated in the 2021 Nobel Prize in Chemistry. This short Perspective aims at providing a brief introduction to the field by first looking on the historical development and the more classical methods and concepts, followed by discussing selected advanced recent examples that opened new directions and diversity within this still growing field.

## Graphical abstract

Asymmetric organocatalysis has developed spectacularly over the last decades, and its general importance was recently highlighted by the 2021 Nobel Prize in Chemistry. This Perspective aims at providing a brief historical introduction to this field, as well as discussing selected advanced recent examples underscoring the diversity and potential of this still growing and vibrant field.

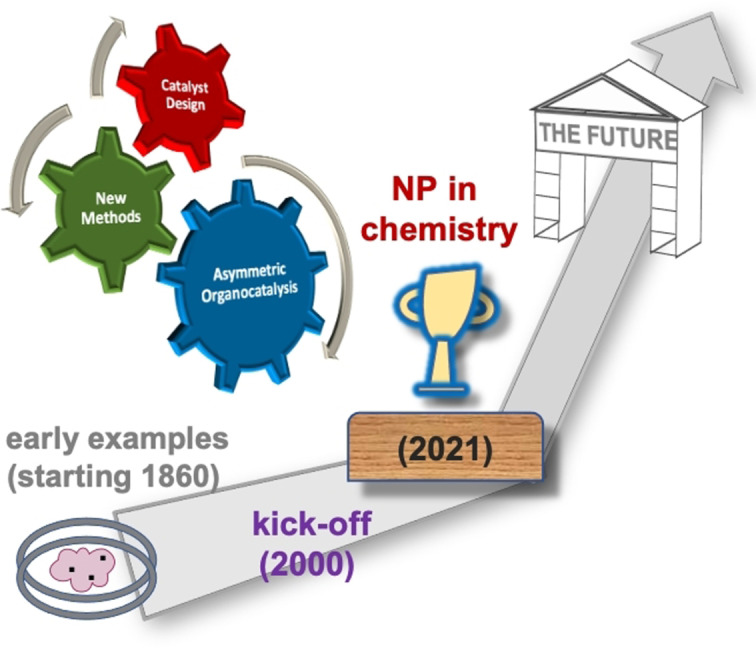

## 1 Introduction and Historical Development

Asymmetric organocatalysis, the use of small chiral organic molecules as catalysts for stereoselective reactions, has been established as the third fundamental pillar in asymmetric catalysis (besides enzymes and metal‐based catalysts) and found its place within the tool boxes of scientists working on purely academic as well as industrial scale projects. Unique concepts and methods have been introduced and the rapid advancement of this field, mainly over the course of the last two decades, has also been publicly recognized by numerous prestigious awards, i. e. the 2021 Nobel prize in chemistry which was awarded to Benjamin List and David MacMillan for “*the development of asymmetric organocatalysis*”. Although the general use of organic compounds as catalysts for organic transformations has been known for more than a century already (vide infra), it was mainly thanks to two seminal independent publications by List, Barbas III, and Lerner^[1]^ and MacMillan's group^[2]^ in 2000 which set the stage for a new trend in organic synthesis. It is also thanks to MacMillan that the classification “*organocatalysis*” has become a generally accepted term for a whole field of research in chemistry, and the number of research groups engaged herein increased tremendously since then.

It should however be emphasized that, although these two milestones from 2000 clearly represent the genesis of modern organocatalysis by initiating one of the biggest hype and competition in organic synthesis over the last decades and by establishing asymmetric organocatalysis as a general field of research, several very important contributions appeared long before. Interestingly, these reports did not receive the broader general attention at the time of their publication and were considered as single individual catalytic methods rather than having the potential to set the foundation for a general catalysis concept. Moreover, these earlier approaches were obviously not considered to belong to the field of organocatalysis, simply because this classification did not exist at the time of their appearance.

**Scheme 1:**
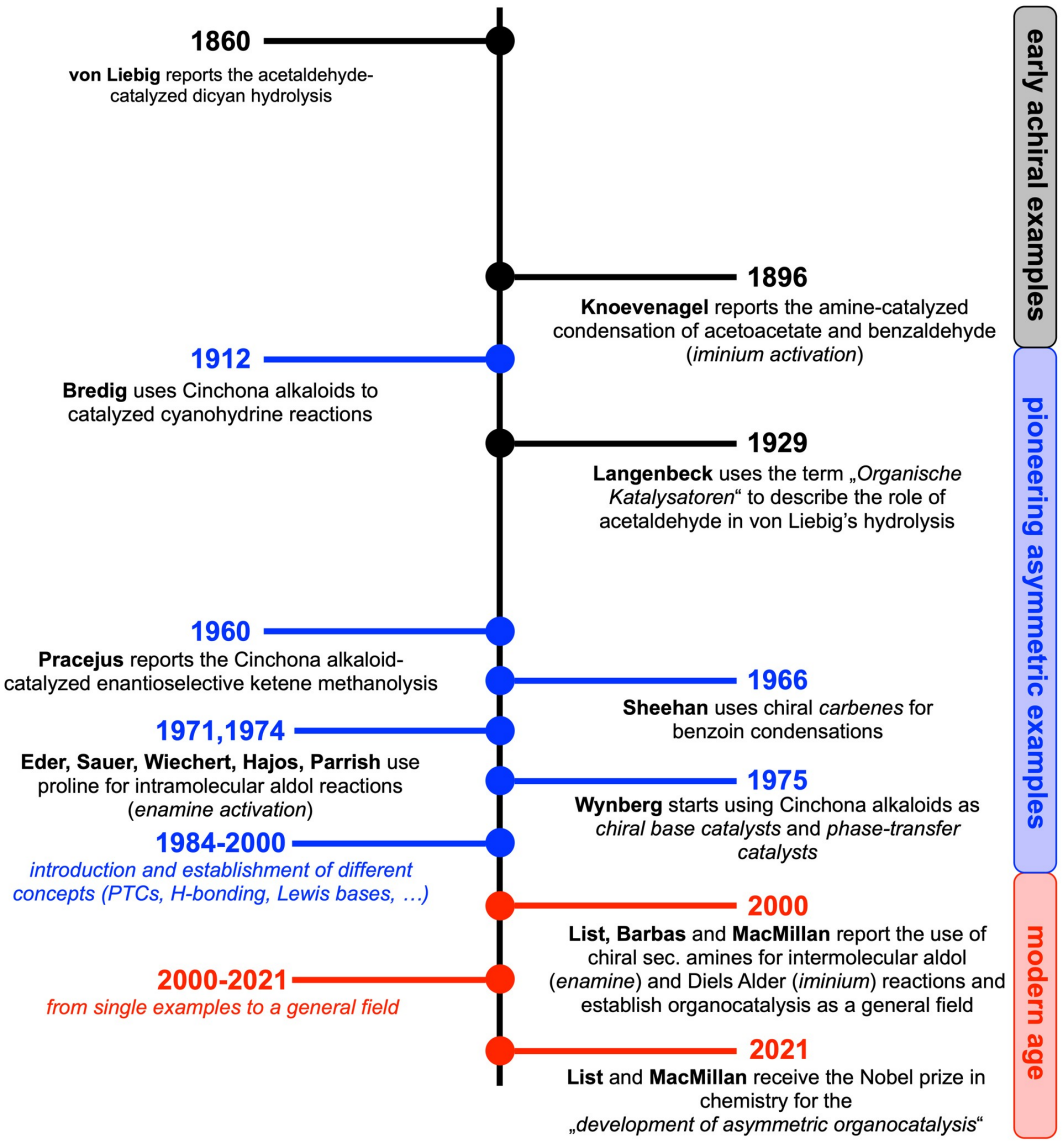
Selected milestones in the historical development of asymmetric organocatalysis.

Historically (Scheme 1), organic molecules have been used as catalysts since Justus von Liebig found that dicyan can be hydrolyzed into oxamide in the presence of an aqueous solution of acetaldehyde in 1860 already,^[3]^ and in 1929 Langenbeck used the German term “*Organische Katalysatoren*” to describe the role of acetaldehyde in Liebig's dicyan hydrolysis.^[4]^ Furthermore, in 1896 Knoevenagel reported the use of secondary amines as catalysts to facilitate the condensation reaction between acetoacetate and benzaldehyde, a reaction proceeding via in situ iminium activation of the aldehyde.^[5]^

Asymmetric organocatalysis can be dated back to the beginning of the last century, when Bredig carried out the addition of HCN to benzaldehyde in the presence of naturally occurring Cinchona alkaloids.^[6]^ With a measurable enantiomeric excess of slightly less than 10 %, this was one of the conceptually groundbreaking reports in this field and in the 1950s Prelog reinvestigated this reaction in more detail.^[7]^ In 1960, Pracejus then reported one of the first highly enantioselective reactions ever, by adding methanol to methyl phenyl ketene in the presence of O‐acetylquinine^[8]^ and in 1966 Sheehan carried out a moderately enantioselective asymmetric benzoin condensation by using chiral carbenes as catalysts.^[9]^ Two milestones which had a lasting influence on modern asymmetric organocatalysis were published in the early 1970s when Eder, Sauer, and Wiechert at Schering^[10]^ and Hajos and Parrish at Hoffmann‐La Roche^[11]^ independently reported the proline‐catalyzed intramolecular aldol cyclization en route to advanced steroid precursors. Remarkably, although Hajos and Parrish considered this reaction to be “*a simplified model of a biological system in which (S)‐proline plays the role of an enzyme*”, this methodology was not developed further for almost 30 years until List, Barbas, and Lerner published their milestone report^[1]^ on the intermolecular proline‐catalyzed aldol reaction proceeding via the nowadays well‐accepted enamine activation mechanism. Several other (mechanistically different) breakthrough reports in the late 1970s, especially by Wynberg's group,^[12]^ and in the early 1980s paved the way for the nowadays established used of Cinchona alkaloids as chiral base catalysts as well as the use of their quaternary ammonium salts as chiral phase transfer catalysts.^[13]^ The 1990s then witnessed the introduction of more and more conceptually different asymmetric strategies catalyzed by chiral organic molecules like, to mention some of the most prominent examples only, Jacobsen's seminal chiral thiourea‐catalyzed Strecker reaction,^[14]^ Miller's use of small peptides as catalysts,^[15]^ the introduction of chiral DMAP derivatives by Fu,^[16]^ Shi's and Yang's chiral ketone‐based oxidation catalysts,^[17]^ Denmark's chiral phosphoramides,^[18]^ and the first report of Maruoka's powerful quaternary ammonium salt catalyst.^[19]^

Altogether, these early reports spectacularly proved the high potential of chiral organic molecules to serve as valuable catalysts for asymmetric transformations but, as already stated above, these methods and catalysts were rather seen as individual developments within their special fields, instead of considering that they may lay the foundation for more generally applicable catalysis concepts which can be applied to a much broader variety of different applications.

It was then thanks to the List and MacMillan that a new era in asymmetric catalysis started when they introduced the generally applicable concepts of enamine and iminium catalysis by using simple chiral secondary amines as catalysts in 2000.[^1^, ^2^] These two reports and the therein presented activation concepts fundamentally changed the way how our community thinks about catalysis and synthesis, not only because of the power of these simple and cheap amine catalysts, but also because of the fact that scientists suddenly realized the sheer potential of using chiral organocatalysts in general. This modern age of organocatalysis attracted numerous research groups and the development of this vibrant and still heavily investigated field over the last two decades has been truly remarkable and last year's Nobel prize in chemistry also proves that asymmetric organocatalysis is nowadays a well‐established and fundamental subdiscipline in chemistry.

## 2 Established Concepts

**Scheme 2:**
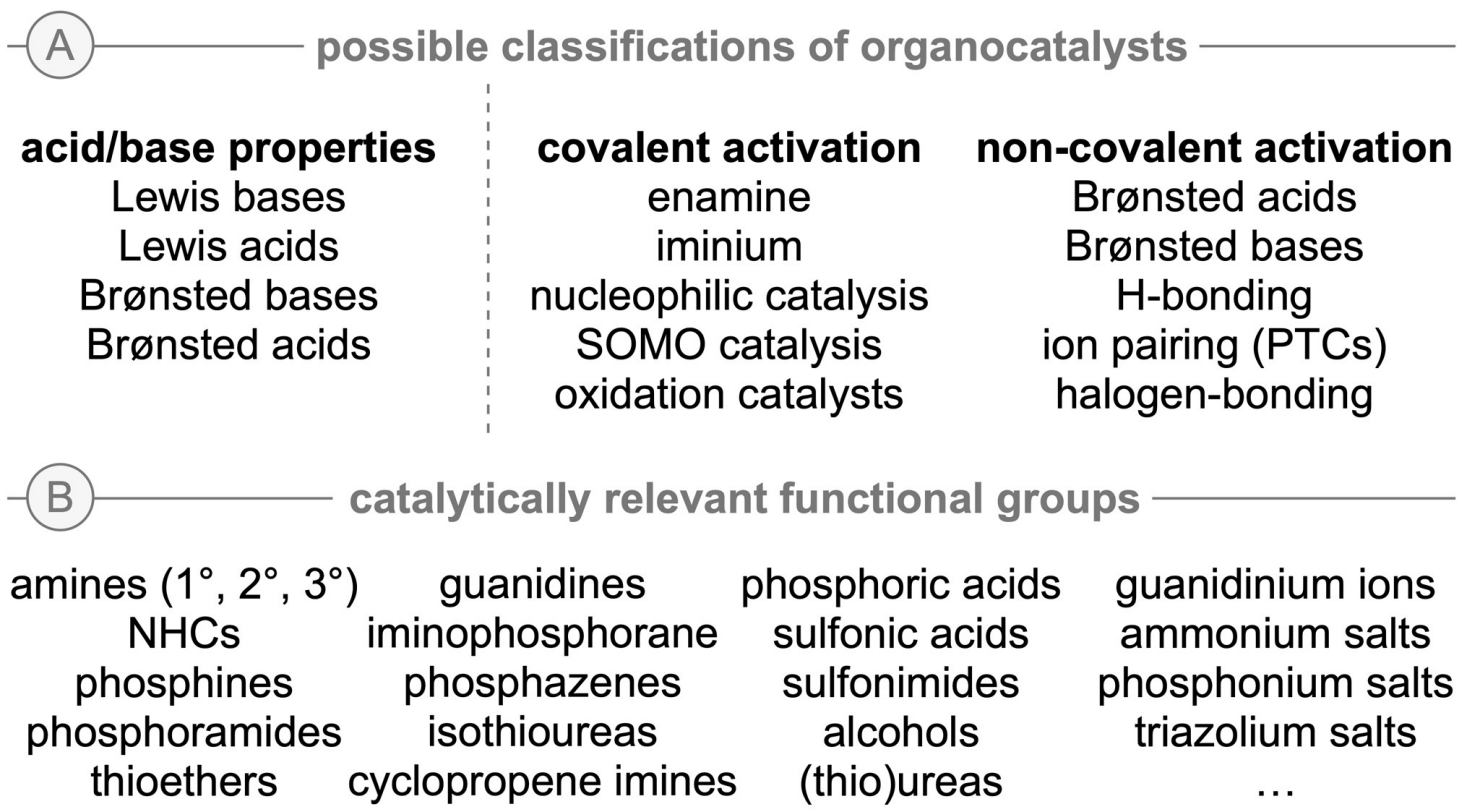
A) Classifications of organocatalysts, and B) established catalytically relevant functional groups.

As can be seen from the early milestones mentioned before, the field of asymmetric organocatalysis has been defined right from the beginning by an enormous variety of conceptually different activation concepts and catalyst classes. By looking at the nowadays established methods in more detail, several ways of classification may be possible (Scheme 2A). One common approach is to classify organocatalysts according to their acid/base properties. Alternatively, one could distinguish them according to their mode of interaction with the starting materials and reagents, that is, by differentiating between catalysts that bind covalently to the substrates and those which activate them by non‐covalent interactions. The diversity of this field can also be highlighted by looking on the nature of the catalysts’ functional groups. A remarkably broad variety of different catalytically competent structural motives are more or less routinely used in (asymmetric) organocatalysis nowadays (Scheme 2B) and the introduction of further powerful catalytically active motives is still a task of high interest and value.

**Scheme 3:**
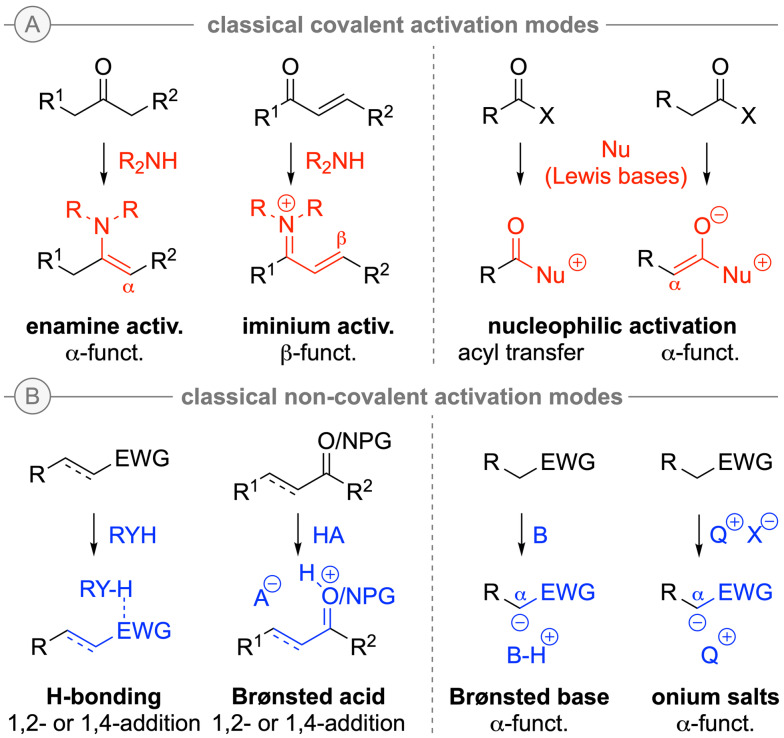
Overview about the most classical organocatalytic activation modes.

Nowadays, such fundamental activation and catalysis modes like enamine and iminium activation, Brønsted acid/base catalysis, nucleophilic Lewis base catalysis, H‐bonding catalysis, and quaternary (amm)onium salt ion pairing phase‐transfer catalysis belong to the established and most commonly used catalysis concepts in the realm of asymmetric organocatalysis. These activation modes allow for a variety of enantioselective transformations (Scheme 3 gives an overview of the most classical activation and reactivity modes) and these nowadays well‐understood concepts have found widespread use not only for academic, but also for industrial applications. Furthermore, besides classical mono‐functional catalysts also bi‐ or multifunctional derivatives (containing different catalytically active groups in one molecule) as well as synergistic catalysis systems (consisting of at least two different single catalysts) have attracted more and more attention over the last years.

In addition to the more classical activation modes (as exemplified in Scheme 3), alternative catalysis concepts and catalyst classes that allow for complementary modes of activation and stereocontrol and which provide an entry to new transformations emerged more recently, and some of the recent trends and developments will be highlighted in the following section. Numerous very illustrative overviews on asymmetric organocatalysis have been published over the last decades^[20]^ and the interested reader is kindly referred to those much more comprehensive articles for specific examples and further details, especially for the more established and nowadays more or less routinely used methods.

## 3 Trends and Recent Developments

The recent trends in organocatalysis aim at expanding the strategies and applicability by combination with other types of emerging catalysis concepts and technologies, as well as designing new substrate activation approaches. Considering the fast and continuous evolution in this research area, the contributions highlighted here are just a small selection of innovative findings and strategies that have inspired the recent advance in the field.

### 3.1 Combination of activation modes – broadening synthetic strategies

**Scheme 4:**
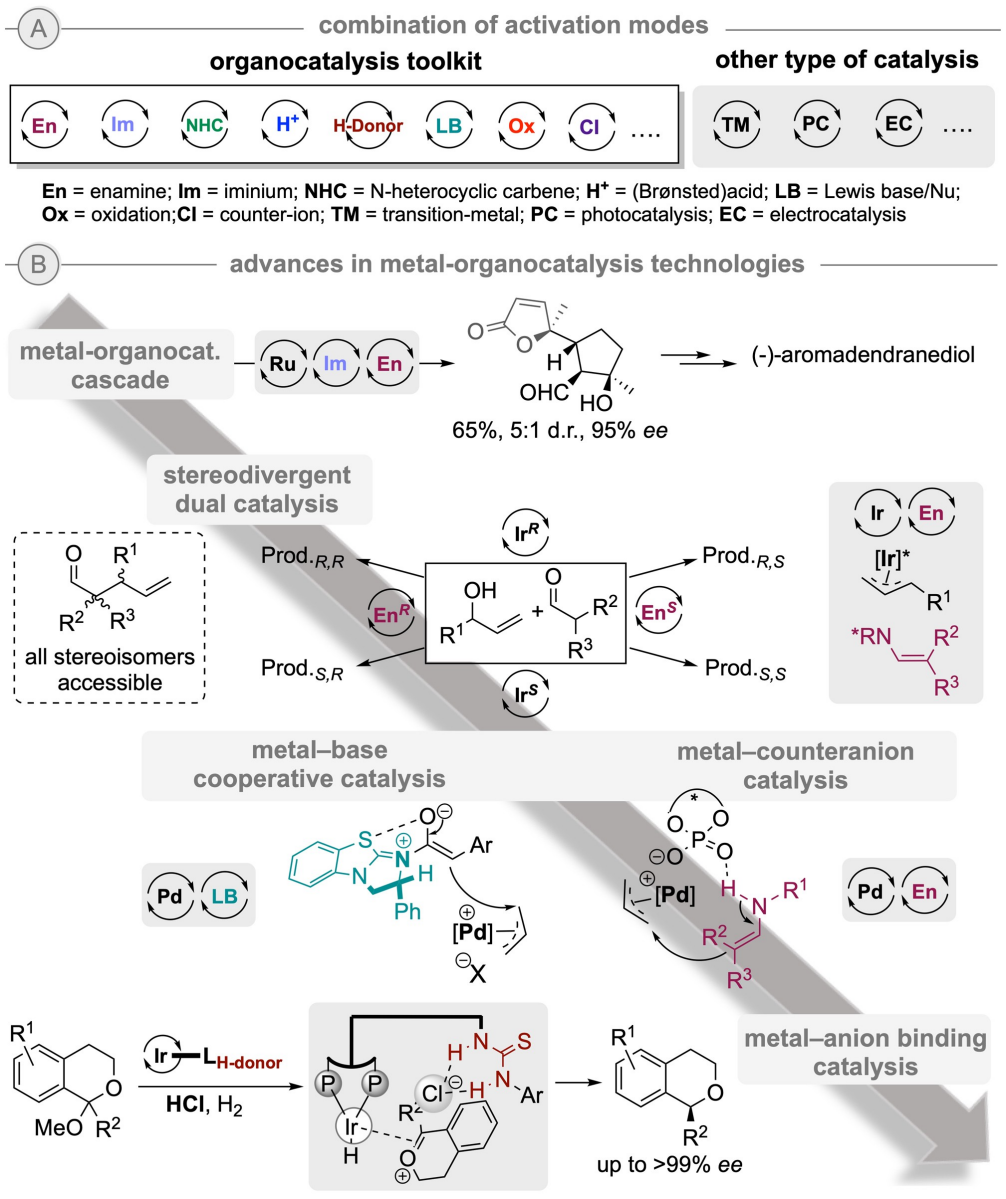
A) Combination of activation modes, and B) selected metal‐organocatalysis technology milestones.

In the past years, the different powerful established activation modes in organocatalysis have been combined in order to achieve more elaborated transformations (Scheme 4A). Hence, synergistic, multicomponent and cascade reactions have been designed,^[21]^ allowing for the construction of products with increased complexity. Inspired by nature, MacMillan's group first showed the potential of this concept by exploiting the dual reactivity of single amine catalysts in cascade iminium‐enamine activation in 2005.^[22]^ Since then, novel strategies for the synthesis of valuable synthetic building blocks and natural products have continuously been developed embracing this approach, from a simple combination (two steps) to more elaborated quadruple or higher catalytic cycles including both covalent and non‐covalent catalysis.

Although initially arisen as alternative or complementary to metal‐catalyzed processes, organocatalysis still often presents some issues due to no or inefficient activation of certain substrate‐types. To overcome this and allow for broader synthetic disconnections and cascade sequences, organocatalysis and transition‐metal (TM) catalysis have been merged to provide new asymmetric processes (Scheme 4B).^[23]^ On the one hand, the incorporation of metal activation in multi‐cascade reactions have already shown a tremendous potential as initially illustrated by MacMillan's group in the synthesis of the terpenoid natural product (‐)‐aromadendranediol employing a Ru‐metathesis – iminium – enamine triple cascade.^[24]^

On the other hand, Carreira and co‐workers designed a fully stereodivergent dual‐catalytic strategy to access all possible stereoisomers in the synthesis of products presenting multiple stereogenic centers, which is still an important challenge in asymmetric catalysis. This was achieved by the right combination of a chiral iridium and a chiral amine catalyst to activate an allylic alcohol and an aldehyde to generate γ,δ‐unsaturated aldehydes with two adjacent stereocenters.^[25]^ Moreover, the group of Snaddon also reported an important work showing the joint forces of the (+)‐benzotetramisole base and XantphosPd catalysts, which enabled a highly enantioselective α‐allylation of acetic acid esters via chiral C1‐ammonium enolates as key intermediates.^[26]^

Furthermore, electrostatic interactions between ionic metallic species and organocatalysts embracing asymmetric counteranion directed catalysis (ACDC) and anion‐binding strategies allow for a fine‐tuning of the catalytic systems. Representative examples include the chiral counter anion – Pd‐catalyzed asymmetric allylation of aldehydes reported by List and co‐workers^[27]^ and the iridium‐catalyzed hydrogenation of in situ formed oxocarbenium ions using a thiourea HB‐donor‐bisphosphine ligand by Zhang's group.^[28]^

Besides these examples in which organocatalysis has been successfully merged with classical metal catalysis concepts, the combination with other currently emerging types of activation modes such as electrocatalysis (EC) or photocatalysis (PC) underline clear future directions.

### 3.2 Organocatalysis’ contribution to photocatalysis

The exponential evolution of photocatalysis has attracted great attention also in the area of organocatalysis.^[29]^ Despite the inherently high reactivity of the photocatalytically generated radicals, which commonly leads to the idea of poor asymmetric control, it offers new venues for the synergetic activation and discovery of unusual reactivities.

**Scheme 5:**
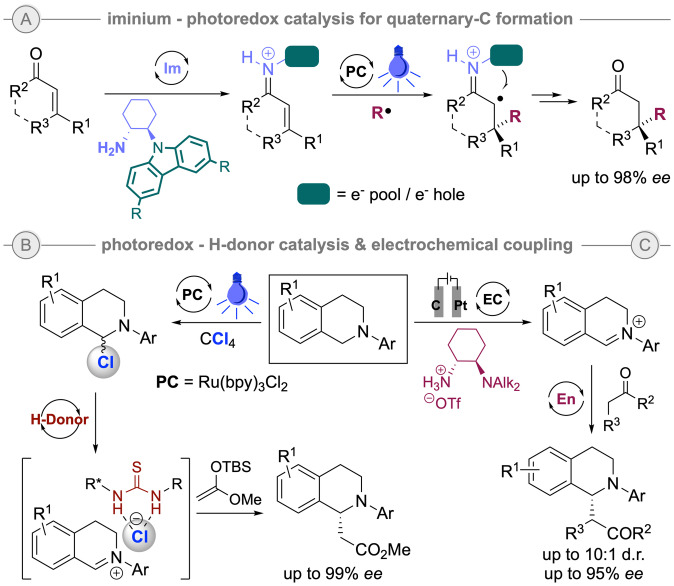
Stepwise one‐pot combination of organocatalysis with metal‐based photoredox catalysis or electrochemical oxidation.

In 2008, Nicewicz and MacMillan first reported the merging of enamine catalysis and metal‐based photoredox catalysis, showing this combination as a new powerful tool to solve some challenging chemical issues as the direct alkylation of aldehydes.^[30]^ Following this inspiring discovery, important contributions appeared in this direction, for which only a few representative early breakthrough examples are highlighted herein. In 2016 Melchiorre's group reported an impressive asymmetric formation of C‐quaternary stereocenters through covalent iminium activation in photocatalysis (Scheme 5A).^[31]^ Alternatively, in the field of non‐covalent organocatalysis, in 2014 Jacobsen and Stephenson showed the efficient sequential combination of photoredox catalysis and asymmetric hydrogen‐donor catalysis via halogen abstraction/anion‐binding catalysis for the functionalization of heterocycles such as THIQs (Scheme 5B).^[32]^ Lately, catalytic electrosynthesis has been introduced as an interesting technique, although it has been less explored than the photoredox variants in oxidative coupling reactions (Scheme 5C).^[33]^

**Scheme 6:**
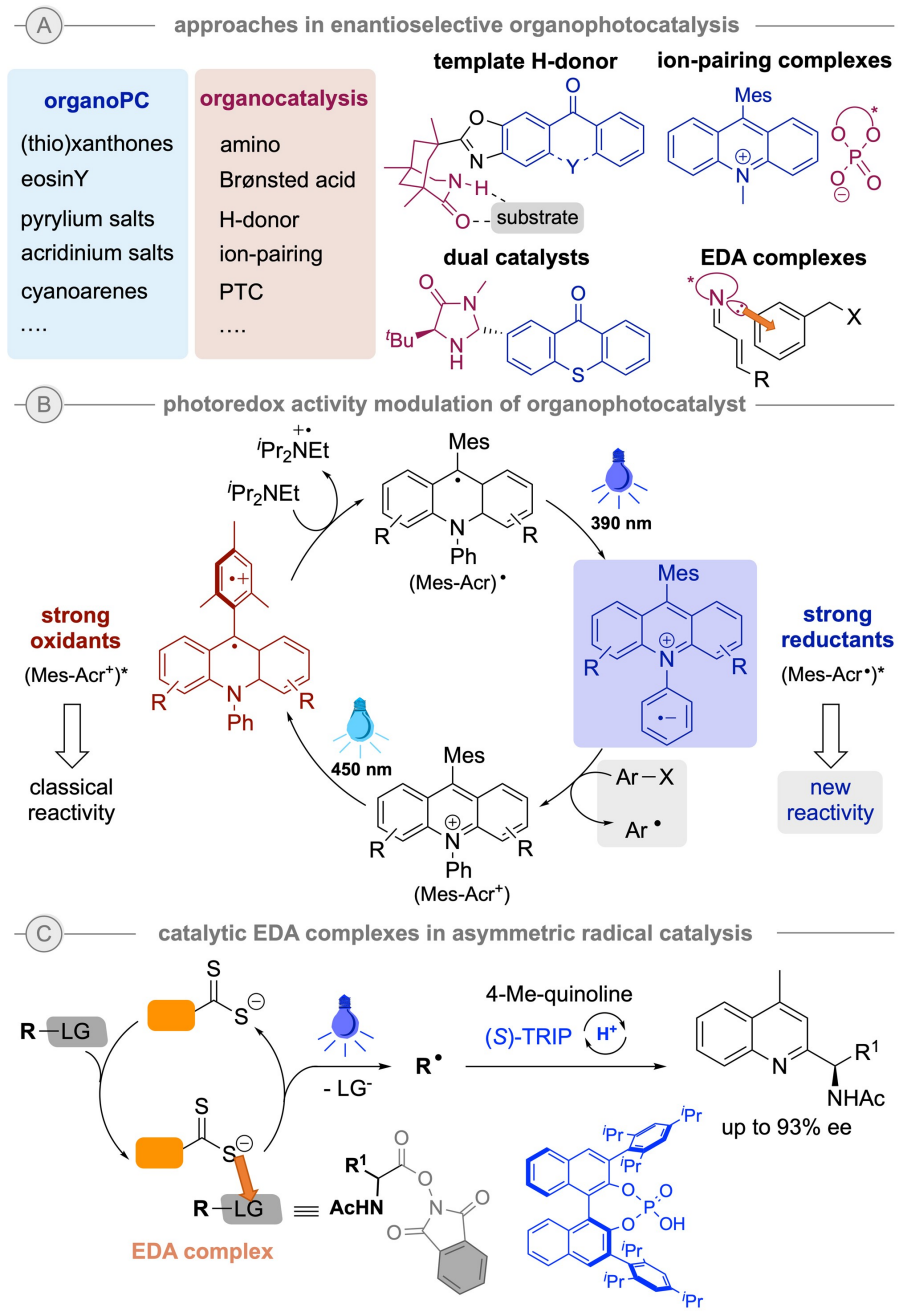
Chiral organophotocatalytic systems and approaches in enantioselective catalysis.

Furthermore, the continuous efforts to develop efficient, potent organic photosensitizers^[34]^ to overcome some of the issues associated to the commonly used costly Ru‐ and Ir‐based photocatalysts have led to the design of innovative catalysts and discovery of exciting, synthetically valuable transformations. Therefore, increasing approaches in enantioselective radical chemistry based on organophotocatalysis have been reported so far, and include not only the combination of organic sensitizers with organocatalysts but also the use of template H‐donor interactions between the catalyst and substrate, dual amino‐organophotocatalysis, ion pairing catalysis with ionic organophotocatalyst or chiral enamine‐substrate EDA complex chemistry (Scheme 6A). A breakthrough example of how to manipulate the intrinsic nature of photoredox catalysts was recently presented by Nicewicz and co‐workers, showing the potential of the still rather unexplored targeted manipulation of the photoredox activity of some sensitizers. Hence, they envisioned a two‐photon excitation of an acridinium salt, well‐known strong oxidants in their excited state, to achieve the Umpolung of its intrinsic reactivity and realize the reduction of arylhalides (Scheme 6B).^[35]^ Although not enantioselective, this report might inspire new chemistry and catalytic system design to be evolve in the near future.

More recently, the discovery of the formation of photoactive electron donor‐acceptor (EDA) complexes formed between the substrate and organic additives or reagents has opened new possibilities, since under these conditions no photocatalyst is required.^[36]^ In this area of research, the recent discovery of Melchiorre and co‐workers on the use of xanthogenate and dithiocarbamate anions as organocatalysts to form photoactive EDA complexes with the substrates and allow for the formation of non‐stabilized C‐ and N‐centered radicals upon visible light irradiation can be highlighted, as this can then be combined with a broader palette of organocatalytic processes (Scheme 5C).^[37]^

### 3.3 Room for creative media

**Scheme 7:**
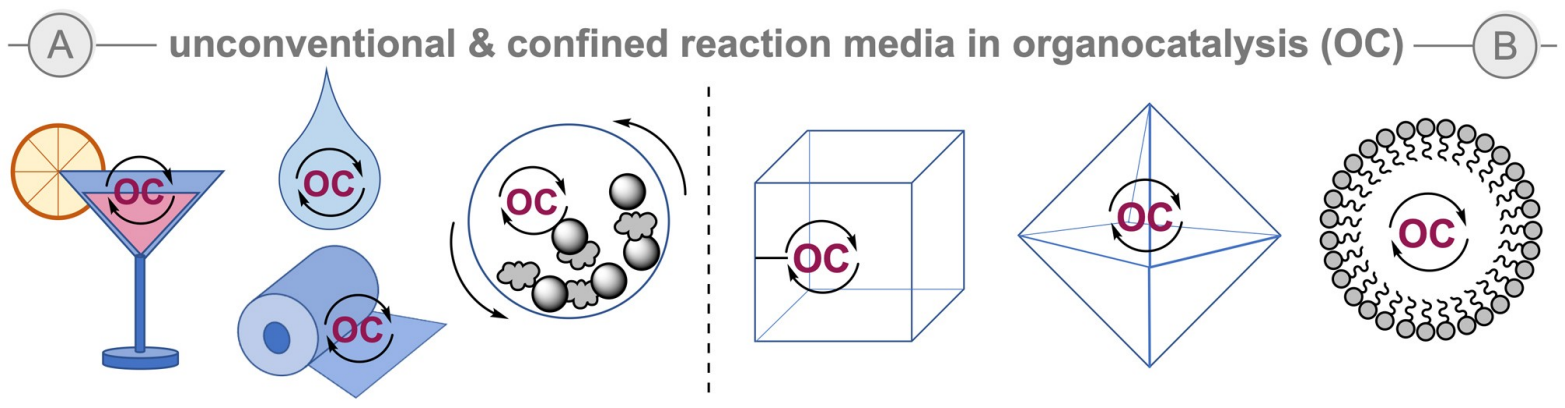
Illustrative non‐classical reaction media in organocatalysis.

Non‐traditional reaction media can be beneficial in organocatalysis. The use of “unconventional” beer and spirituous, ionic liquids, water or brine as solvents often lead to improved outcomes, while microwave, ultrasound or solvent‐free conditions (e. g. ball milling) can notable accelerate these reactions (Scheme 7A). Scientists in the field are continuously showing a great creativity in the reaction media employed. Among those, it is worthy to highlight the use of organotextile catalysis introduced by List and co‐workers in 2013,^[38]^ in which a variety of catalysts were immobilized in textile nylons and showed high enantiocontrol while their excellent stability and recyclability makes this approach very attractive for potential industrial applications.

Furthermore, confined catalysts and media have also been envisioned for the development of novel organocatalytic processes (Scheme 7B).^[39]^ Thus, the use of enzyme mimicking catalysts with confined active sites, heterogenized organocatalytic decorated metal‐organic frameworks (MOFs) and cages^[40]^ or use of micellar environment^[41]^ provides a unique confined reaction media that allows for unusual conformational restricted geometries and/or highly selective processes.

### 3.4 Emerging catalyst designs, activation approaches and interaction toolkits

**Scheme 8:**
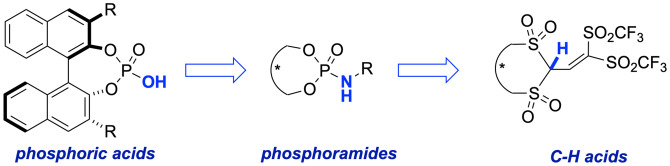
Exemplary design‐evolution of chiral Brønsted acids.

The design of new catalyst structures is a continuous and dynamic activity occurring in the field of organocatalysis. A clear example is the development of Brønsted acid catalysts that allowed enhanced activities or the discovery of innovative transformations. Hence, from the initial BINOL‐based phosphoric acids, which have long dominated this field,^[42]^ to phosphoramides with improved acidity to the more recently designed C−H acids first reported by List and co‐workers in 2016 (Scheme 8).^[43]^

Moreover, in the last years, the tendency has moved from a defined single key activation mode toward a more enzyme‐like use of multiple interactions. In this regard, different combinations of all types of non‐covalent interlinkages have gained tremendous importance, allowing for sophisticated activation strategies to reach boosted stereoselectivities.

**Scheme 9:**
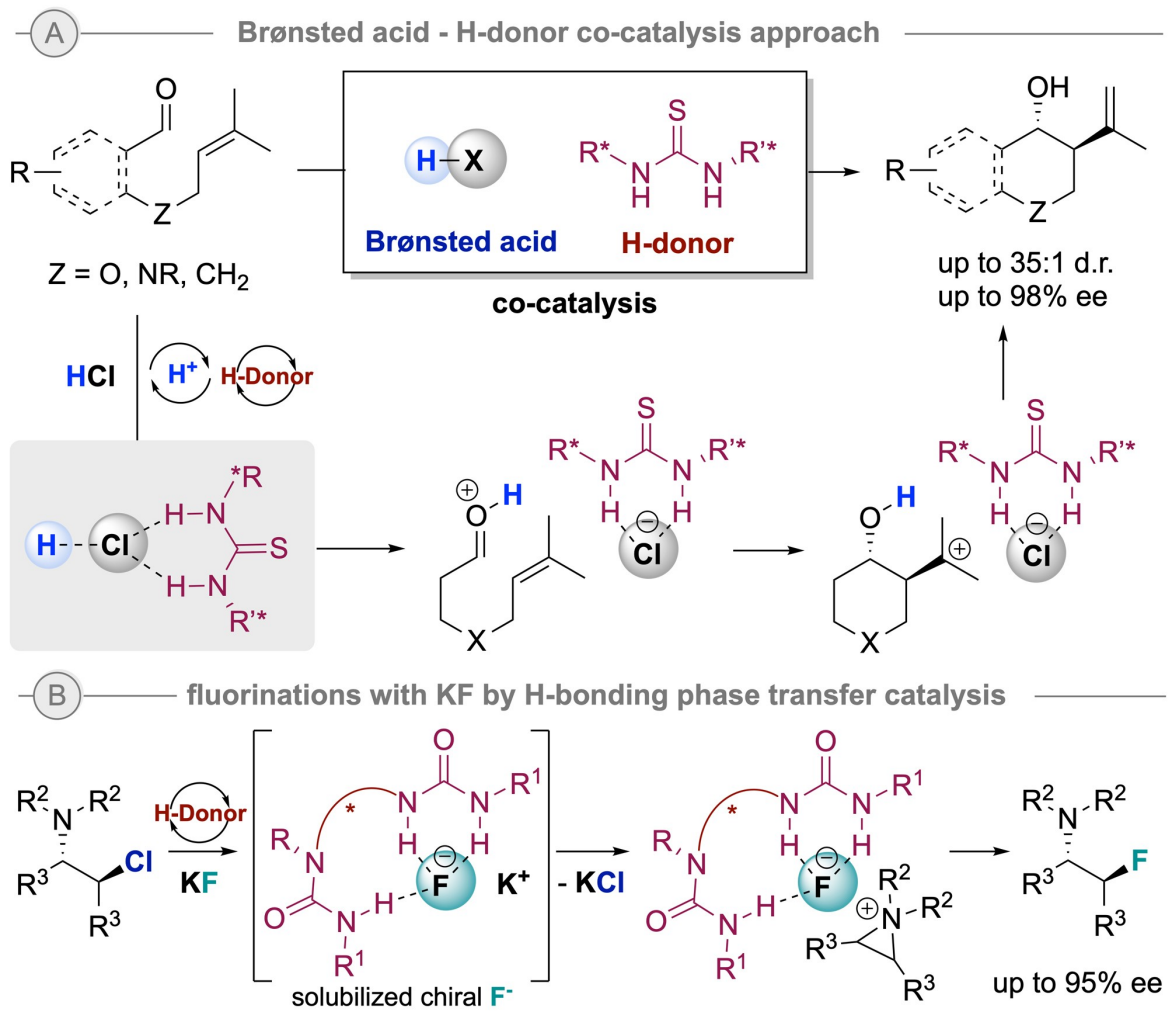
Selected innovative activation approaches: A) Brønsted acid – HB‐donor co‐catalysis, and B) H‐bonding PTC for enrolling KF as nucleophile.

Another trend in organocatalysis is the design of innovative activation approaches that might lead to enhanced or different reactivities that could not be easily achieved by other means. An excellent example is the HB‐donor ‐ acid co‐catalysis approach introduced by the group of Jacobsen.^[44]^ Hence, catalytic chiral H‐donor species can modulate the activity of Lewis and Brønsted acids as demonstrated in a recent example for the thiourea/HCl co‐catalyzed asymmetric Prins cyclization shown in Scheme 9A.^[45]^ A further recent breakthrough approach was reported by the group of Gouverneur, who achieved the activation of simple highly insoluble KF as fluoride source by hydrogen bonding phase‐transfer catalysis using bis‐ureas for the enantioselective synthesis of β‐fluoroamines (Scheme 9B).^[46]^

**Scheme 10:**
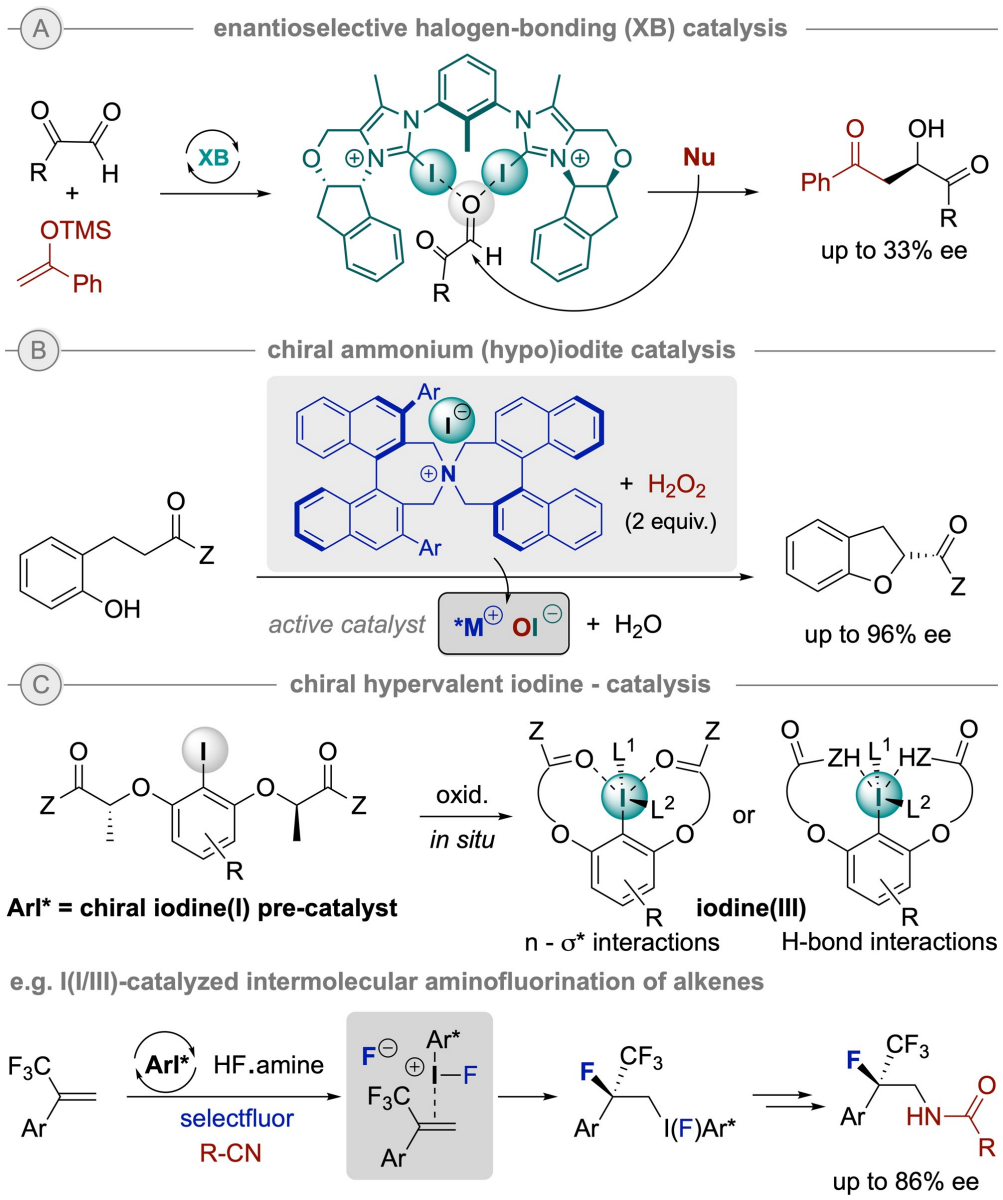
Different approaches in enantioselective iodine‐based catalysis.

The exploitation of other types of non‐covalent interactions such as halogen bonding (XB), as well as chalcogen (ChB) and pnictogen bonding (PnB) through the surface electrostatic potential hole (the so‐called σ‐hole) of organo‐halide, ‐chalcogen and ‐pnictogen species has attracted recent attention.^[47]^ These interactions are more directional but usually stronger than the parent, classical hydrogen bonding and they have the potential of not only being more efficient in some transformations but allow for new reactivities. However, to date only moderate enantiomeric inductions have been achieved in reactions based on solely this type of interactions as for the iodoimidazolium‐based XB‐donor catalyzed enantioselective Mukaiyama aldol reaction reported by Huber and co‐workers (Scheme 10A),^[48]^ while more promising results have been monitored when joining other types of interactions.

Iodine‐based redox catalysis has gained increased attention in the past few years and recently emerged as a potent activation mode in the organocatalysis toolkit.^[49]^ On the one hand, in 2010 Ishihara and co‐workers introduced an inspiring seminal example on the use of chiral ammonium salts paired with inorganic iodine‐derived oxo‐acids such as (hypo)iodites for an asymmetric oxidative cycloetherification reaction.^[50]^ The active species were easily formed from the corresponding iodide salt by treatment with H_2_O_2_ (Scheme 10B). On the other hand, more attention has been set on the development of chiral hypervalent iodine catalysts. Among chiral hypervalent reagents, aryliodines(III) have become privileged species for iodine enantioselective catalysis.^[51]^ These can be smoothly generated in situ by oxidation of the corresponding iodine(I) precursor (Ar*I). The most prominent chiral catalyst backbone relies on a resorcinol core bearing two chiral lactic‐derived side chains, which can be easily fine‐tuned to stabilized the I(III) center by n‐σ* or H‐bond interactions, while providing an appropriate, confined catalyst active site (Scheme 10C). Hence, this strategy has led to a number of innovative methodologies, from which the recent work of Gilmour's group can be highlighted as this approach has allowed for the intermolecular aminofluorination of olefins with inverted‐regioselectivity.^[52]^

The combination of iodine(I/III) redox catalysis with electrochemistry by in situ (re)generation of hypervalent iodine represents an appealing new track in iodine‐based organocatalysis. Hence, this strategy has been shortly introduced by Powers and co‐workers for efficient oxidation reactions such as oxidative C−H amination of arenes,^[53]^ which also promises future possible extensions to enantioselective catalysis.

### 3.5 Data intensive approaches and machine‐learning assisted organocatalysis

The rational design of optimal catalytic systems for a targeted transformation has always been in the heart of catalysis. Besides trial‐and‐error strategies, including speeding‐up high‐throughput methods, data‐intensive approaches for mechanistic elucidation have become more popular. The generation of large data, its organization and analysis of structural parameters that affect the reaction outcome can afford an improved trends overview and facilitate catalyst optimization. A notable example of this approach for chiral anion catalysis was reported by Toste and Sigman in 2015.^[54]^ Finally, the contemporary evolving machine‐learning strategies^[55]^ in organic chemistry for the prediction of reactivity have also a great potential for their implementation in the design or appropriate selection of organocatalysts for specific targeted reactions. Indeed, in 2019 Denmark and co‐workers already successfully used this approach to predict the enantiomeric induction of chiral phosphoric acids in an asymmetric addition reaction of thiols to imines by averaged steric occupancy (ASO) data analysis.^[56]^

## 4 Future Directions

Asymmetric organocatalysis has developed spectacularly over the last two centuries and it is unquestionably that future developments will open new directions and possibilities allowing applications and transformations that are not feasible yet.

One scientific aspect that will be of uttermost importance, at least in the authors’ opinion, will be the introduction of new catalytically competent motives and functional groups, as well as the establishment of alternative chiral backbones. It has been well demonstrated in the past that the introduction of new catalysts leads to new transformations and concepts, and it is therefore without doubt that the rational design of new catalysts will still be fundamental for the future advancement of the field. While catalyst design was so far mainly a trial‐and‐error approach, we are confident that the current progress in computational methods, i. e. machine learning techniques, the increasing mechanistic understanding of organocatalytic reactions, and the establishment of reliable concepts for the parametrization of catalysts, will lead to a more rapid and straightforward identification of new catalysts in the (closer) future. In addition, this shall also lead to the introduction of powerful synergistic multicatalytic approaches, as well as concepts where two catalytically complementary catalysts may self‐assemble/self‐organize in the reaction mixture, thus leading to powerful synergistic multifunctional catalyst systems.

With respect to future applications, we are convinced that (asymmetric) organocatalysts have the potential to open further new directions in catalysis. So far, the main focus in the field was on the utilization and synthesis of moderately complex smaller organic molecules and the use of organocatalytic methods in total syntheses of complex molecules mainly focused on the synthesis of smaller earlier building blocks. However, it can be envisioned the future development of efficient organocatalysis tools for the highly selective late‐stage functionalization of complex molecules. This will contribute significantly to the general development of more efficient (also protecting‐group‐free) approaches that should also allow for biorthogonal applications in complex reaction media. In addition, organocatalysis will most likely also become more routinely used in the activation of small molecules like O_2_, CH_4_, CO_2_, etc. In this case, the value will not be on stereocontrol but rather on activity. Considering the outstanding potential of a variety of different catalytically competent organic functional groups to facilitate reactions where other catalysis concepts may fail, we are very much confident that organocatalysis (maybe even in combination with complementary activation modes) will play an important role in small‐molecule activation soon.

## Disclaimer

The opinions expressed in this publication are the view of the author and do not necessarily reflect the opinions or views of the *European Journal of Organic Chemistry*, the Publisher, Chemistry Europe, or the affiliated editors.

## Conflict of interest

The authors declare no conflict of interest.

## Biographical Information

*Olga García Mancheño was born in Cuenca, Spain, in 1976 and studied chemistry at University Autonomous of Madrid, where she obtained her Ph.D. in 2005 in the group of Prof. Juan Carlos Carretero. After a postdoctoral stay in the group of Prof. Carsten Bolm at RWTH Aachen, she carried out her habilitation at the WWU Münster. In 2013, she was appointed at the University of Regensburg and, in 2017, at the WWU Münster as professor for organic chemistry. Her main research interests cover the developing new, efficient methodologies in organic chemistry, with especial focus on the design of novel catalytic systems and their application in homogenous catalysis, including photocatalysis and asymmetric non‐covalent and anion‐binding catalysis*.

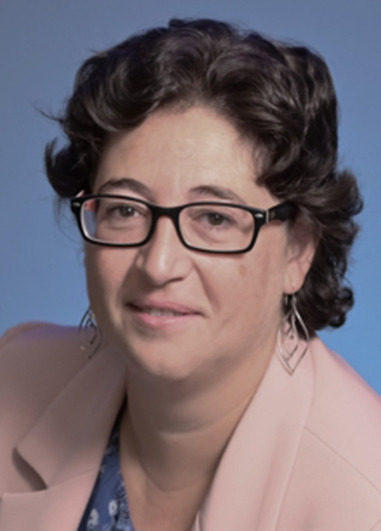

## Biographical Information

*Mario Waser was born in Steyr, Austria in 1977 and studied chemistry at JKU Linz, Austria, where he obtained his Ph.D. in 2005 in the group of Prof. Heinz Falk. After a postdoctoral stay with Prof. Alois Fürstner (Max‐Planck Institut für Kohlenforschung, Mülheim, Germany), he spent two years as an R&D chemist working for DSM. In 2009, he started his independent career at JKU Linz and in 2014 he obtained his habilitation (venia docendi) and became Associate Professor. In 2020, he was promoted to Full Professor for Organic Stereochemistry and in 2021 he was appointed as the Head of the Institute of Organic Chemistry at JKU. His main research interests are on the design and application of asymmetric organocatalysts (i. e. quat. ammonium salt‐based ion pairing catalysts) and on the development of asymmetric organocatalytic synthesis methods*.